# Integrated microbiomic and proteomic profiling reveals distinct ocular surface molecular and microbial landscapes in dry eye after SMILE surgery

**DOI:** 10.3389/fcimb.2026.1858069

**Published:** 2026-07-15

**Authors:** Li Zhang, Peng Chen, Pengfei Han, Huizhe Fu, Bin Sun

**Affiliations:** 1 Shanxi Medical University, Taiyuan, China; 2 Shanxi Eye Hospital Affiliated to Shanxi Medical University, Taiyuan, China; 3 Shanxi Aier Eye Hospital (Shanxi), Taiyuan, China

**Keywords:** multi-omics, ocular surface microbiome, postoperative dry eye disease, small incision lenticule extraction (SMILE), tear proteomics

## Abstract

**Objectives:**

The aim of this study is to investigate the microbial and proteomic features of the ocular surface following small incision lenticule extraction (SMILE) surgery and to elucidate their association with the development of postoperative dry eye disease (DED).

**Methods:**

This study comprised a prospective cohort for baseline assessment and a cross-sectional postoperative assessment. We enrolled two sets of participants: a preoperative cohort (PG) consisting of patients scheduled for SMILE; and a postoperative cohort,consisting of patients who had undergone SMILE. The postoperative cohort was further categorized into non-dry eye group (NDEG) and dry eye group (DEG) groups based on examination. Conjunctival swabs and tear samples were collected for 16S rRNA gene sequencing (V3-V4 region) and label-free quantitative proteomics, respectively.

**Results:**

A total of 52 subjects were included. Microbiome analysis revealed significantly lower α-diversity (Chao1, Shannon) in the DEG than in the PG and/or NDEG (*p* < 0.05). β-diversity analysis showed significant structural differences between the DEG and both the PG and NDEG. Staphylococcus and Corynebacterium were enriched in the DEG, while commensals such as Clostridia and Bacteroidota showed higher relative abundances in the NDEG. DEG exhibited specific activation of stress pathways (NF-κB signaling, macroautophagy) and a bidirectional dysregulation of the coagulation-complement system, a pattern suggestive of a potential “thromboinflammation”-like state. Weighted gene co-expression network analysis delineated three axes: an overactive “Metabolism-Stress Axis”, and impaired “Homeostatic Regulation Axis” and “Orderly Repair Axis” in the DEG.

**Conclusions:**

In this cross-sectional analysis, the presence of DED after SMILE is associated with a distinct shift in the ocular surface microbiome towards a pro-inflammatory state and a concomitant breakdown of host proteomic homeostasis. The observed correlation between microbial dysbiosis and dysregulation of host pathways, particularly the coagulation-complement system, suggests a potential integrated mechanism for postoperative dry eye. These findings identify candidate biomarkers for risk prediction and new therapeutic targets.

## Introduction

Refractive surgery provides patients with the opportunity to achieve independence from spectacles and contact lenses (Mysore and Krueger, 2015). In recent years, femtosecond laser small incision lenticule extraction (SMILE) has become one of the mainstream procedures for refractive correction due to its minimally invasive nature, rapid visual recovery, and stable efficacy (Han et al., 2019; Wan et al., 2025). However, postoperative dry eye disease (DED) remains a common complication affecting patient satisfaction (Kim et al., 2019; Wong et al., 2019). Its clinical manifestations are diverse, including ocular dryness, foreign body sensation, asthenopia, redness, frequent blinking, and intermittent visual blurring (Nair et al., 2023). Although compared to traditional laser *in situ* keratomileusis (LASIK), SMILE typically results in milder and shorter-duration dry eye symptoms due to less extensive corneal nerve damage, significant inter-individual variation in postoperative dry eye response persists among patients (Wan et al., 2025). This heterogeneity points to biological mechanisms beyond anatomy and physiology that likely influence postoperative DED susceptibility.

The pathogenesis of postoperative DED is complex, involving multiple factors such as corneal nerve damage, decreased tear film stability, and ocular surface inflammatory responses (Wong et al., 2019; Liu et al., 2021). Recent advances in microbiomic and proteomic technologies have provided novel perspectives for exploring the essence of ocular surface diseases at a microsystemic level. More and more studies found that a low-biomass yet dynamically balanced microbial community exists on the healthy ocular surface (Doan et al., 2016; Garza et al., 2021). Through interactions with the host immune system, they play a crucial role in maintaining local homeostasis and defending against pathogens. Dysbiosis of the ocular surface microbiome is closely associated with various inflammatory conditions, including DED and blepharitis (Ozkan and Willcox, 2019; Liang et al., 2021; Zhao et al., 2025). Concurrently, the tear fluid proteome serves as a sensitive indicator reflecting ocular surface physiological and pathological states. Its dynamic changes can reveal processes, such as inflammatory activation, tissue repair, and stress responses (Khazaei et al., 2021). As a significant intervention to the ocular surface microenvironment, SMILE surgery may be associated with differences in microbial composition compared to preoperative individuals. This could be associated with changes in local immune and inflammatory status through microbe-host interactions, which may be reflected by the expression profiles of the tear proteome. Consequently, systematically comparing compositional differences between groups in the ocular surface microbiome and tear proteome before and after surgery holds promise for identifying molecular and microbial correlates of the pathogenesis of postoperative DED from microecological and molecular perspectives, providing a new theoretical basis for understanding clinical variability and advancing personalized postoperative management.

This prospective cohort study employs integrated 16S rRNA gene sequencing and label-free quantitative proteomics to systematically compare the conjunctival microbiome and tear proteomic profiles among preoperative patients, postoperative patients without DED, and postoperative patients with DED after SMILE. We aimed to identify DED-associated microbial taxa and proteins, and to explore associations between microbial community composition and host proteomic responses in individuals with established postoperative DED. This work lays the experimental and data foundation for future construction of biomarker-based models for early identification and risk prediction of DED.

## Methods

### Ethics and patients enrollment

This study was approved by the Ethics Committee of Taiyuan Aier Eye Hospital (Approval No. EYETYYY-20251117-1). Participants were myopic patients scheduled to undergo SMILE surgery at the hospital between February and May 2025. All participants provided written informed consent. Basic demographic and clinical information was obtained from all enrolled individuals.

### Surgery

This study comprised a cross-sectional design with two independent cohorts: a preoperative group (PG) representing the baseline state, and a postoperative cohort recruited cross-sectionally. While this design identifies associations, it cannot establish causality. Inclusion criteria for all participants were: (1) age 18–45 years; (2) a diagnosis of myopia with clinical assessment confirming suitability for corneal laser refractive surgery; (3) either scheduled for PG or having undergone (postoperative cohort) SMILE. Exclusion criteria included keratoconus, active ocular surface infection, lagophthalmos, eyelid malposition or deformity, pregnancy or lactation, a history of allergy or keloid predisposition, or any history of psychiatric disorders, autoimmune diseases, or significant dysfunction of major organs.

### Sample collection, storage, and processing

#### Standardization and pre-collection precautions

All collections were performed by a single experienced ophthalmologist. To ensure comparability across groups, the right eye was uniformly sampled for all participants; the left eye was used only when the right eye was unavailable (two cases, recorded). Critically, no topical anesthetic (e.g., proparacaine, tetracaine) was applied before sampling, as anesthetics can reduce detectable microbial diversity and inhibit bacterial growth.

#### Conjunctival swab collection and preservation

Conjunctival swabs were collected from the inferior conjunctival fornix on the day of preoperative examination (PG group) and at the 3-month postoperative visit (NDEG and DEG groups). The patient was asked to look upward, and a sterile flocked swab was gently rotated over the inferior fornix for approximately 3–5 seconds, with a separate sterile swab used for each eye. Immediately after collection, the swab tip was cut into a sterile cryotube, transported on ice (4 °C), and transferred to –80 °C storage within 2 hours of collection. DNA extraction was performed within 6 months of storage, and samples were never thawed before extraction.

#### Tear collection and teflex tear mitigation

Tears (15–30 µL) were collected using 5 µL glass microcapillary tubes. The patient was asked to tilt the head slightly to the opposite side and look laterally. The capillary tip was gently placed at the lateral tear meniscus of the lower lid margin, without touching the bulbar conjunctiva or lid margin, to minimize stimulation. Tears were drawn by capillary action. To minimize reflex tearing, the following precautions were taken: (a) all collections were performed by the same experienced operator; (b) patients were allowed to blink intermittently, and the capillary was withdrawn during blinks; (c) if sudden excessive tearing occurred, collection was paused for 5 minutes before a new attempt. Given that postoperative dry eye patients had reduced basal tear secretion, reflex tearing was less likely, and the collected fluid predominantly represented basal tears. After collection, tears were expelled into a sterile microcentrifuge tube, kept on ice for no longer than 30 minutes, and then stored at –80 °C. Proteomic analysis was performed within 3 months of storage.

#### Inter-facility transfer and freeze-thaw management

All samples (swabs and tears) underwent only one freeze–thaw cycle (directly from –80 °C to extraction/digestion). Dry ice shipping was used for all inter-facility transfers. Swabs were extracted within 6 months and tears were analyzed within 3 months of collection.

### Microbiome analysis

Following DNA extraction from 52 samples, the 16S rRNA gene V3-V4 region was amplified. After merging and quality control of raw reads, DADA2 was used for denoising to generate amplicon sequence variants (ASVs). Alpha diversity (Chao1, Shannon index) and beta diversity (using Bray-Curtis distance) were calculated based on the ASV table. Intergroup comparisons of alpha diversity were performed using Tukey’s Honest Significant Difference test. Beta diversity analysis was conducted using Principal Coordinates Analysis (PCoA) and Non-metric Multidimensional Scaling (NMDS) based on Weighted and Unweighted Unifrac distances. Permutational multivariate analysis of variance (PERMANOVA; ADONIS test) was employed to assess statistical differences in community structure between groups. Linear discriminant analysis Effect Size (LEfSe) was utilized to identify significantly differential microbial taxa between groups. Subsequently, random forest modeling was employed to construct classification models and identify key discriminatory species across taxonomic levels. The performance of these models was evaluated and compared using receiver operating characteristic (ROC) curves, with feature importance quantified by Mean Decrease Accuracy and Mean Decrease Gini.

### Proteomic analysis

Tear samples were processed and subjected to liquid chromatography-tandem mass spectrometry (LC-MS/MS) analysis. Label-free quantification was used to obtain protein expression profiles. Differentially expressed proteins (DEPs) were screened based on set thresholds (|log_2_FC| > 1, *p* < 0.05). Identified DEPs were subsequently analyzed for Gene Ontology (GO) functional annotation, Kyoto Encyclopedia of Genes and Genomes (KEGG) pathway enrichment, and Hallmark gene set analysis. To identify clusters of co-expressed proteins and explore their functional relevance, fuzzy c-means clustering via the Mfuzz algorithm was performed. Spearman correlation analysis was performed between the relative abundances of significantly enriched microbial genera and the expression levels of differentially expressed proteins.

### Data availability

The data reported in this paper have been deposited in the China National Center for Bioinformation/Beijing Institute of Genomics, Chinese Academy of Sciences (https://ngdc.cncb.ac.cn, accession no. PRJCA056136). The authors declare that the main data supporting the findings are available within this article. The other data generated and analyzed for this study are available from the corresponding author upon reasonable request.

## Results

### Baseline characteristics

A total of 52 patients were enrolled, comprising 21 in the PG, 11 in the NDEG, and 20 in the DEG. The mean age was 23.5 ± 5.5 years old, and the ratio of male was 51.9% (27/52). The spherical equivalent ranged from -6.25 to -2.25 D. The highest ablation depth was 146 μm and the lowest ablation depth was 60 μm. Tear meniscus height ranged from 0.19 to 0.46 mm. The highest central corneal thickness (CCT) was 610 μm, found in NDEG. The longest Non-invasive tear film breakup time (NIBUT) First and NIBUT average were 24 sec, while the shortest NIBUT first and NIBUT average were 2.9 sec and 5.7 sec, respectively. However, no significant differences were observed in baseline characteristics among the three groups (*p* > 0.05) (**Table 1**).

**Table 1:** Baseline characteristics of enrolled patients.

Clinical characteristics	Total	DEG (N = 20)	NDEG (N = 11)	PG (N = 21)	*p*
Age, years	23.5 ± 5.5	20.6 ± 3.2	24.9 ± 4.6	25.4 ± 6.7	0.012
Sex, n (%)					0.274
Female	25 (48.1%)	7 (35%)	7 (63.6%)	11 (52.4%)	
Male	27 (51.9%)	13 (65%)	4 (36.4%)	10 (47.6%)	
Spherical equivalent, D	-4.9 ± 1.0	-4.9 ± 1.3	-5.1 ± 0.7	-4.7 ± 0.9	0.529
Central corneal thickness (CCT), μm	545.5 ± 24.6	552.1 ± 22.5	551.9 ± 31.3	535.8 ± 20.1	0.062
Ablation depth, μm	107.0 ± 18.4	109.4 ± 21.6	107.4 ± 11.5	104.5 ± 18.6	0.705
Flap thickness, μm	118.3 ± 5.5	119.0 ± 4.5	118.2 ± 6.0	117.6 ± 6.2	0.731
Non-invasive tear film breakup time (NIBUT) First, sec	12.0 ± 6.6	12.2 ± 6.5	10.4 ± 6.3	12.6 ± 7.1	0.667
NIBUT average, sec	15.5 ± 4.9	15.3 ± 4.7	14.8 ± 5.4	16.2 ± 5.0	0.74
Tear meniscus height (TMH), mm	0.3 ± 0.1	0.3 ± 0.1	0.3 ± 0.1	0.3 ± 0.1	0.406
Redness index	0.9 ± 0.2	0.9 ± 0.2	1.0 ± 0.2	0.9 ± 0.3	0.831
Ocular Surface Disease Index (OSDI)	4.0 ± 3.6	4.6 ± 4.4	4.4 ± 2.5	3.2 ± 3.1	0.447
Strip Meniscometry Tube (SMTube), mm	7.3 ± 1.5	7.4 ± 1.3	7.0 ± 1.2	7.5 ± 1.9	0.669
Corneal fluorescein staining (FL)	85.2 ± 11.5	87.9 ± 11.0	82.3 ± 10.6	84.2 ± 12.3	0.387

### Microbiome analysis results

**Figure 1:**
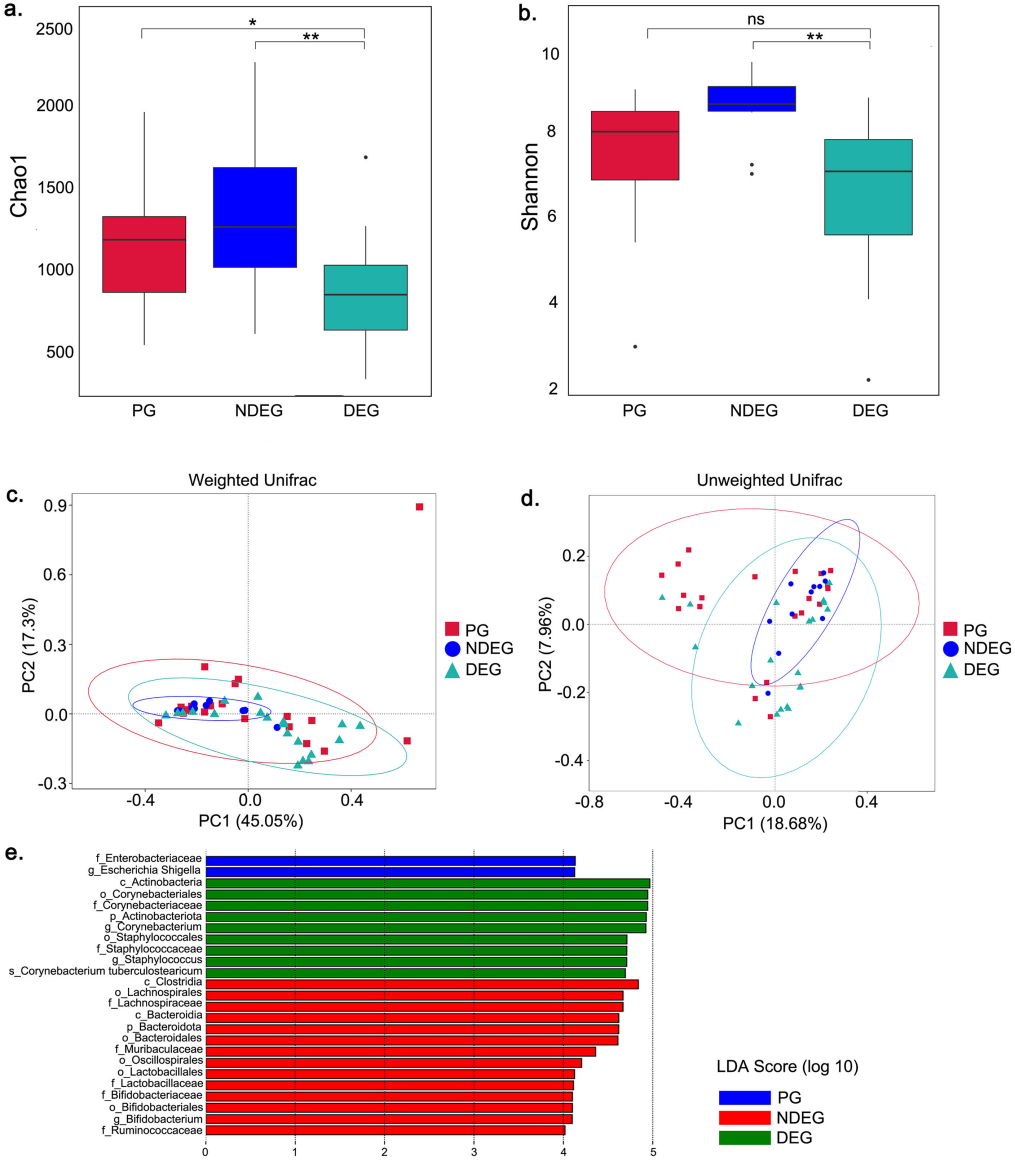
Comparison of microbial communities among the three groups. Alpha diversity was assessed using the Chao1 **(a)** and Shannon **(b)** indices. Beta diversity was visualized through PCoA plots based on Weighted **(c)** and Unweighted **(d)** Unifrac distances. Differential analysis between groups was performed using LDA, with results shown as LDA scores and enriched taxa **(e)**. *P<0.05, **P<0.01, ns, not significant.

Alpha diversity analysis revealed that the Chao1 index in the DEG was significantly lower than that in both the PG and NDEG (*p* < 0.05). The Shannon index was also significantly lower in the DEG compared to the NDEG (*p* < 0.05). No significant difference in alpha diversity was found between the NDEG and PG (**Figure 1a, b**). These results indicate that the group of patients diagnosed with DED after SMILE surgery exhibited a significant decrease in both species richness and diversity of the ocular surface microbial community. In contrast, patients without DED postoperatively had alpha diversity measures that were not significantly different from those in the PG. This suggests that lower ocular surface microbial community diversity in the postoperative period is associated with the presence of DED.

We further assessed differences in overall microbial community structure. PCoA and NMDS results showed extensive overlap in the distribution of samples from the three groups, indicating no pronounced differentiation in overall microbiota composition among groups (**Figure 1c, d**; **Supplementary Figure 1**). Samples from the NDEG exhibited a more clustered distribution pattern, suggesting relatively consistent microbiota structure among individuals within this group. Conversely, samples from the DEG were more dispersed, indicating greater inter-individual variation in microbial composition within this group. PERMANOVA analysis further revealed statistically significant differences in community structure between the DEG and the PG (R² = 6.3%, *p* = 0.005) and between the DEG and the NDEG (R² = 10.2%, *p* = 0.001), with the difference from the NDEG being particularly significant. The difference in community structure between the NDEG and the PG was not significant (R² = 5.4%, *p* = 0.066). These results suggest that the overall structure of the ocular surface microbiota was similar between the PG and NDEG, whereas patients with DED exhibited a community composition that differed significantly from both other groups and higher individual heterogeneity, with their microbiota structure being notably distinct from that of patients without DED.

At the phylum level, the conjunctival microbiota across the three groups shared a similar core composition, predominantly consisting of *Actinobacteria*, *Firmicutes*, *Proteobacteria*, and *Bacteroidota* (**Supplementary Figure 2**). However, LEfSe analysis revealed significantly differential enrichments among the three groups at finer taxonomic levels (LDA score > 4.0, *p* < 0.05; **Figure 1e**). The DEG was significantly enriched in genera often associated with dysbiosis or opportunistic infections, particularly *Staphylococcus* and *Corynebacterium* (including the species *Corynebacterium tuberculostearicum*). Conversely, the NDEG was significantly enriched in taxa typically considered commensal or beneficial, primarily represented by the class *Clostridia*, the phylum *Bacteroidota* (specifically the order *Bacteroidales* and family *Muribaculaceae*), and the order Bifidobacteriales. The PG exhibited a unique microbial signature characterized by significant enrichment of the *Escherichia-Shigella* genus.

This clear postoperative differentiation pattern was further validated by supervised machine learning. Using different numbers of top species features, random forest model was used to classify PG versus DEG (**Supplementary Figure 3**). In the training set, the area under the receiver operating characteristic curve (AUC) ranged from 76.89% to 88.24%, with the optimal performance achieved using 30 top species features (AUC = 88.24%). Remarkably, all models demonstrated perfect discriminatory power in the independent test set, with an AUC of 100%. These findings indicate that the microbiota composition in postoperative patients differs from that in preoperative patients, and that the presence of DED is specifically associated with higher abundance of potentially pro-inflammatory bacteria, the occurrence of DED is specifically associated with the enrichment of potentially pro-inflammatory or pathogenic bacteria, whereas favorable postoperative outcomes are linked to the enrichment of typical commensal communities.

### Proteomic analysis results

**Figure 2:**
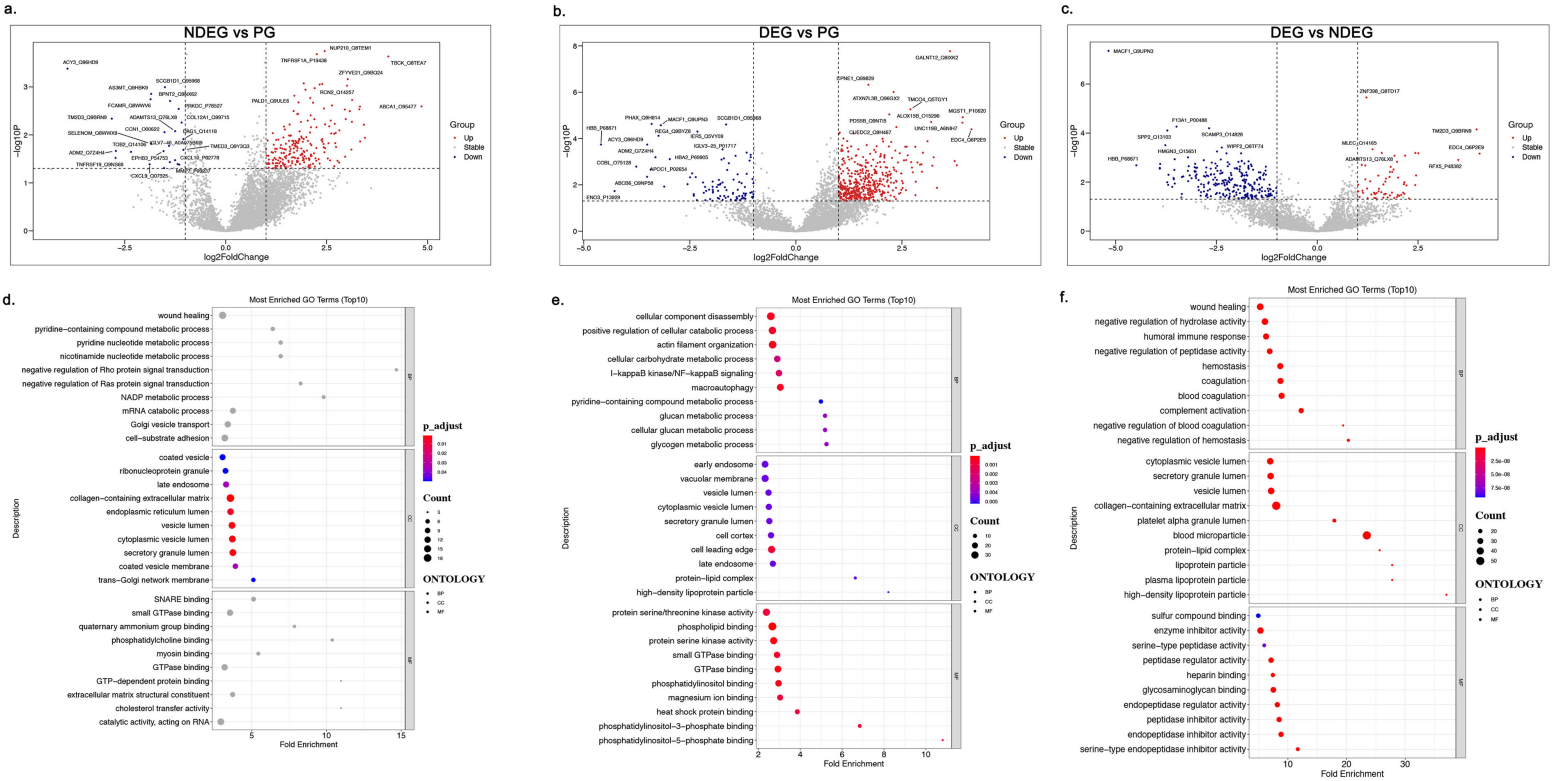
Differential expression and functional enrichment analysis. **(a–c)** Volcano plots of differentially expressed proteins between groups. **(d–f)** The corresponding top 10 enriched GO terms for each comparison (DEG vs PG, NDEG vs PG, DEG vs NDEG, respectively).

A total of 5433 quantifiable proteins were identified across all samples from the three groups. Comparative analysis revealed 560 DEPs between the DEG and the PG, with 467 significantly upregulated and 93 significantly downregulated (**Figures 2a-c**). Between the NDEG and the PG, 239 DEPs were identified (215 upregulated, 24 downregulated). Direct comparison between the DEG and the NDEG yielded 317 DEPs (62 upregulated, 255 downregulated). Overall, compared to preoperative levels, the tear proteome in both postoperative groups differed significantly from that in the PG, predominantly characterized by higher expression levels of many proteins, indicating that SMILE surgery itself triggers a substantial proteomic response. Furthermore, the DEG exhibited widespread suppression of protein expression compared to the NDEG.

Both postoperative groups showed significant upregulation of pathways related to vesicular transport, secretory functions, and post-transcriptional regulation. This included enrichment of cellular components such as coated vesicles, late endosomes, and secretory granule lumens, as well as enhanced GTPase binding, SNARE binding, and catalytic activity acting on RNA (**Figure 2d, e**; **Supplementary Figures 4**, **5**). Concurrently, pathways related to excessive extracellular matrix organization, chemokine activity, and other inflammatory and repair processes were commonly suppressed postoperatively, reflecting an early regulatory mechanism to prevent over-repair (**Supplementary Figure 5**). However, the NDEG and DEG exhibited fundamental divergence in their proteomic response patterns. Characteristically, the NDEG, building upon the shared response, actively and significantly downregulated pathways like extracellular matrix organization and chemokine activity, overall presenting an orderly, controlled repair state. In contrast, the DEG showed higher expression of proteins in stress and remodeling pathways, including cellular component catabolism, macroautophagy, and I-κB kinase/NF-κB signaling. This was accompanied by significant upregulation of cellular carbohydrate metabolic processes and the PI3K-AKT-mTOR signaling pathway, indicating a state of persistent catabolism, autophagic clearance, and inflammatory stress.

Further direct comparison between the DEG and NDEG revealed dry eye-specific molecular imbalance features (**Figure 2f**; **Supplementary Figure 6**). Notably, both upregulated and downregulated DEPs in the DEG *vs.* NDEG comparison were simultaneously and significantly enriched in coagulation, complement activation, and their negative regulation pathways. Hallmark analysis showed significant downregulation of the complement and coagulation pathways in the DEG, suggesting a bidirectional dysregulation of the coagulation-complement system characterized by concurrent abnormal activation and compensatory inhibition, forming the molecular basis of “thromboinflammation”. Additionally, the DEG exhibited extensive reprogramming of post-translational modification pathways, such as glycoprotein biosynthesis and N-glycan processing. These results corroborate the aforementioned comparisons, confirming that while dry eye patients share the basic postoperative repair program, their homeostatic regulatory networks have specifically collapsed.

**Figure 3:**
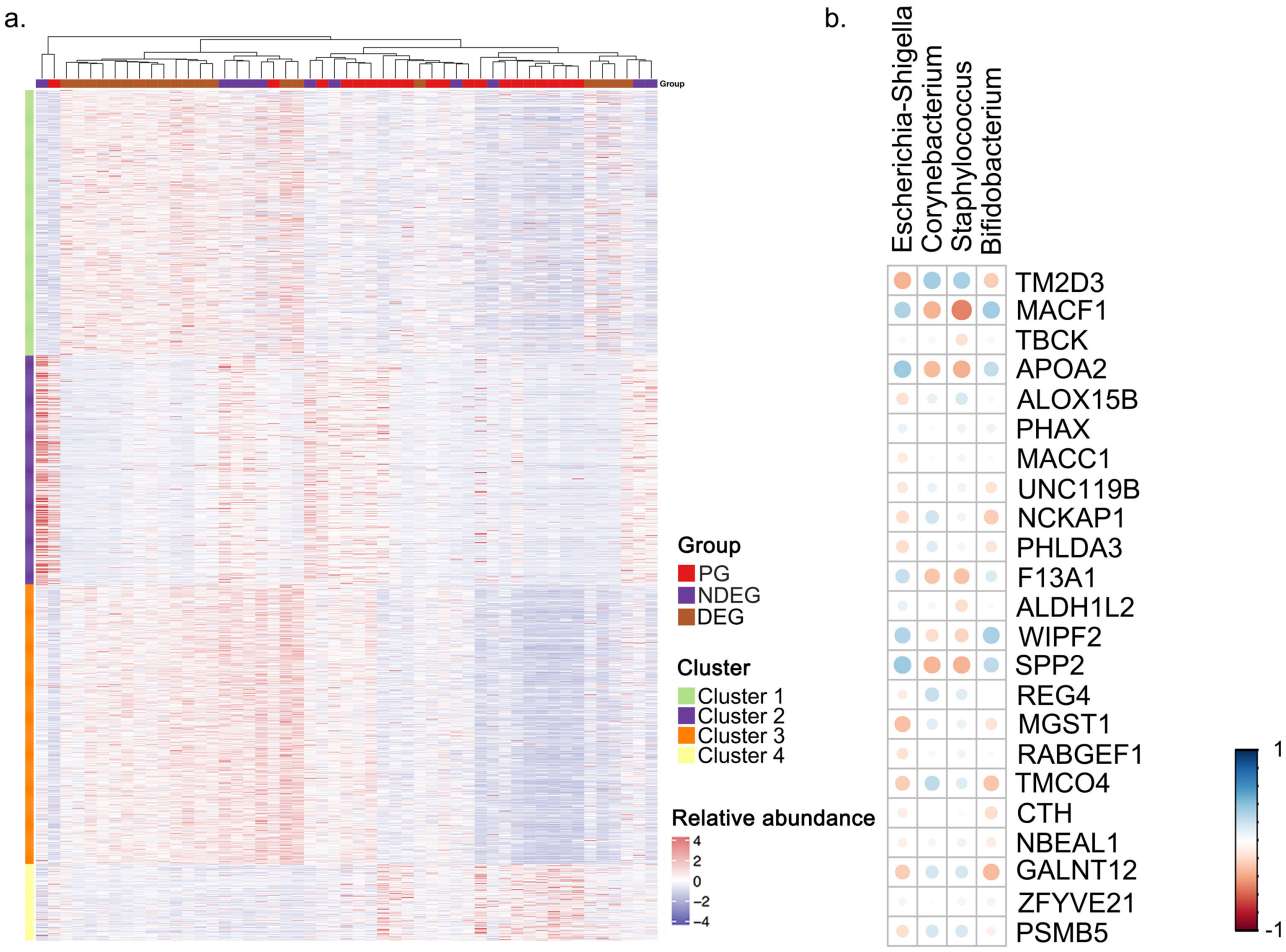
Systems-level integration of proteomic and microbiome data. **(a)** Heatmap of co-expressed protein clusters identified by Mfuzz analysis across the three groups. **(b)** Spearman correlation results between differentially abundant microbial taxa and differentially expressed proteins.

To integrate these findings at a systems level, fuzzy c-means clustering via Mfuzz was performed across all samples, identifying four core protein clusters with expression patterns significantly associated with clinical phenotypes (**Figure 3a**). Go functional analysis of these four core protein clusters (**Supplementary Figure 7**) revealed that Cluster 1 (high expression in DEG) and Cluster 3 (high expression in DEG) were enriched in carbohydrate metabolism/NF-κB signaling and autophagy/lysosomal function, respectively, together constituting an abnormally activated “Metabolism-Stress Axis”. Cluster 2 (low expression in DEG) was highly enriched in coagulation-complement system regulatory proteins. and its lower expression in the DEG suggests functional impairment of the “Homeostatic Regulation Axis”. Cluster 4 (low expression in DEG) was enriched in extracellular matrix organization and cytokine signaling proteins, and its downregulation indicates damage to the “Orderly Repair Axis”. This co-expression model elucidates that the occurrence of postoperative DED stems from the abnormal activation of the “Metabolism-Stress Axis” coupled with the functional failure of the “Homeostatic Regulation Axis” and the “Orderly Repair Axis”, which was associated with the collapse of ocular surface microenvironment homeostasis in the DEG.

ROC analysis (**Supplementary Figure 8**) based on differentially expressed proteins between groups revealed that the distinction between the DEG and the PG showed the highest diagnostic performance, with an AUC of 0.93 (95% CI: 0.855-1), while it was difficult to distinguish between PG and NDEG (AUC = 0.65 (95% CI: 0.431-0.86)) or between DGE and NDEG (AUC = 0.73 (95% CI: 0.522-0.942)).

### Correlation between microbial taxa and protein expression

To explore potential interactions between the ocular surface microbiome and host proteomic responses, we performed correlation analysis between differential abundant microbial genera and differentially expressed proteins. Significant correlations (*p* < 0.05 with |r| > 0.3) were identified between key microbial taxa enriched in different clinical groups and several host proteins (**Figure 3b**), suggesting potential statistical links between microbial abundance and host molecular responses. In the DEG, *Staphylococcus* and *Corynebacterium*, both enriched in this group, exhibited strong positive correlations with TM2D3 (*Staphylococcus*: r = 0.35, *p* = 0.013. *Corynebacterium*: r = 0.33, *p* = 0.019), a protein implicated in cellular stress and metabolism. Conversely, they showed negative correlations with MACF1 (*Staphylococcus*: r = −0.35, *p* = 0.012. *Corynebacterium*: r = −0.49, *p* = 0.00024), a cytoskeletal regulator involved in tissue integrity. In contrast, in the NDEG, *Bifidobacterium* (representative of the enriched *Bifidobacteriales* order) was positively correlated with MACF1 (r = 0.31, *p* = 0.029) and APOA2 (r = 0.35, *p* = 0.011), a lipid metabolism protein, while showing a negative correlation with TM2D3 (r = −0.35, *p* = 0.012). These correlation patterns reinforce the notion that the postoperative ocular surface microbiome is functionally linked to host proteomic states.

## Discussion

This cross-sectional study provides the first integrated description of interconnected microbiomic and proteomic changes associated with postoperative DED after SMILE, offering a novel systems biology perspective for generating hypotheses about its pathogenesis. Our findings in a cross-sectional cohort indicate that the presence of postoperative DED is correlated with a characteristic pattern involving microbial dysbiosis, aberrant host stress responses, and signatures of homeostatic network dysregulation, rather than the result of some single factor.

This study found that patients with DED after SMILE had significantly lower alpha diversity in their ocular surface microbiome, coupled with enrichment of potentially pro-inflammatory genera (e.g., *Staphylococcus*, *Corynebacterium*), and their community structure demonstrated higher inter-individual heterogeneity. This aligns with previous views linking ocular surface dysbiosis to DED (Zegans and Van Gelder, 2014; Andersson et al., 2021; Schlegel et al., 2023; Zhang et al., 2025). Notably, patients with good postoperative recovery showed higher relative abundances of commensal communities represented by *Clostridia*, *Bacteroidota*, and *Bifidobacteriales* (Budden et al., 2017; Zilliox and Bouchard, 2023). In addition, their alpha diversity showed no significant difference from preoperative levels, and the overall microbiota structure was more consistent. This suggests that the ability to maintain microbial diversity and a commensal state postoperatively may be closely related to susceptibility to DED. The microbial changes in this postoperative dry eye condition are similar to those in the nasal microbiome of patients with chronic sinusitis (Foster et al., 2020; Huang et al., 2022). PERMANOVA analysis further indicated that, despite overlap in the overall microbial structure of the three groups, statistically significant differences existed between the DEG and both the PG and NDEG. This implies that the occurrence of postoperative DED may not stem from a wholesale restructuring of the microbiota but rather from the different relative abundances of specific taxa and their interaction with the host microenvironment. Additionally, the random forest model constructed based on microbial species features provides strong evidence for the crucial role of the microbiome in distinguishing postoperative states.

Echoing the microbial changes, proteomic analysis revealed deeper host molecular disturbances. the tear proteome of postoperative DED patients differed significantly from that of the NDEG and PG in several respects. Compared to those with good recovery, DED patients not only activated a “Stress-Metabolism Axis” centered on cellular catabolism, autophagy, and NF-κB signaling, but also exhibited functional impairment of key regulatory networks maintaining ocular surface homeostasis. This pattern of heightened stress signaling coupled with a loss of regulatory control mirrors findings in complex immune-mediated diseases such as Sjögren’s syndrome, where proteomic studies have similarly identified distinct, subtype-associated dysregulation in NF-κB signaling, oxidative stress responses, and cellular metabolism (Sun, 2017; Berry et al., 2024). The most prominent evidence was the bidirectional dysregulation of the coagulation-complement system: both activation pathways and negative feedback inhibition pathways of this system were simultaneously enriched in the DEG, while the overall Hallmark signal was downregulated. This strongly suggests that in the dry eye state, the coagulation and complement systems are abnormally activated, and the body’s compensatory inhibitory feedback is insufficient to restore balance, leading to a chronic “thromboinflammatory” microenvironment on the ocular surface (Santos et al., 2023). The collapse of this finely tuned network is a core molecular feature distinguishing the DEG from the NDEG. Mfuzz fuzzy clustering analysis further integrated these scattered molecular events into a systemic model of synergistic imbalance, characterized by the abnormal activation of the “Metabolism-Stress Axis” and the functional failure of both the “Homeostatic Regulation Axis” and the “Orderly Repair Axis”, thereby clearly delineating the pathological pathway from molecular perturbation to the functional phenotype of DED.

The most insightful finding of this study lies in the potential correlation and consistency between microbiome and proteome alterations. On one hand, pro-inflammatory microbes enriched in the DEG (e.g., *Staphylococcus*) might serve as triggers driving local immune-inflammatory responses and activating the host’s “Metabolism-Stress Axis” (Bachert et al., 2012). On the other hand, the homeostatic dysregulation (e.g., coagulation-complement system disorder) and repair impairment (e.g., downregulated extracellular matrix organization) revealed by the host proteome may further alter the ocular surface microenvironment, creating conditions favorable for the colonization and proliferation of specific microbial taxa, thereby forming a vicious cycle of dysbiosis, inflammatory activation, tissue damage, and microenvironment change. This bidirectional interaction between microbes and the host jointly promotes the dysbiosis of postoperative ocular surface microbiota and the progression of DED.

Furthermore, our correlation analysis between differential abundant microbial genera and key host proteins provides direct evidence for this microbiome-proteome interplay. We found that *Staphylococcus* and *Corynebacterium*, enriched in the DEG, were positively correlated with TM2D3 (a protein upregulated in the DEG and implicated in cellular stress) and negatively correlated with MACF1 (a cytoskeletal regulator) downregulated in the DEG. Conversely, in the NDEG, *Bifidobacterium* showed a negative correlation with TM2D3 and a positive correlation with both MACF1 and APOA2, a lipid metabolism protein. These correlations suggest that postoperative microbial shifts are not merely associative but may functionally contribute to the divergent proteomic profiles observed between dry eye and non-dry eye patients, reinforcing the concept of a coordinated microbial–host molecular axis in postoperative ocular surface homeostasis.

This study has some limitations. First, as an observational study, it reveals strong associations, but causal relationships require further validation through animal models or *in vitro* experiments. Second, while the sample size sufficed for preliminary exploratory analysis, a larger cohort would help verify the generalizability of the biomarkers identified. Furthermore, the most significant limitation is the cross-sectional design. By comparing separate groups of patients (preoperative, postoperative with DED, postoperative without DED) rather than following the same individuals longitudinally, this study cannot establish a temporal sequence or infer causality. For example, we cannot determine whether the observed microbial dysbiosis preceded and contributed to DED, or whether the DED state (or its treatment) induced the microbial and proteomic changes. Therefore, all discussions of mechanisms, triggers, or drivers are strictly hypothesis-generating. Our use of terms like “associated with” and “correlated with” reflects this associative nature. Future prospective, self-controlled studies with longitudinal sampling are essential to distinguish surgical effects from disease-related changes and to test the causal hypotheses generated by this work. Future prospective self-controlled studies will help more accurately distinguish the causal relationships between surgical effects and disease states. Lastly, the study time points were relatively fixed, unable to dynamically capture the complete trajectory of postoperative microbial and proteomic evolution.

In summary, this study constructs an integrative framework for the pathogenesis of DED after SMILE from microecological and molecular systems dimensions. Our findings suggest that postoperative DED is characterized by a cross-sectional signature of ocular surface homeostatic imbalance that is associated with both microbial dysbiosis and dysfunction of host homeostatic networks, such as the coagulation-complement system. While causality cannot be determined from this study, the identification of these candidate biomarkers and pathways generates the hypothesis that microbes and host factors interact in DED pathogenesis, such as the coagulation-complement system. In the future, interventions targeting the commensal microbiota enriched in patients with good recovery, or modulating key molecules within the “Homeostatic Regulation Axis”, may become novel strategies for preventing and treating SMILE-induced postoperative DED. Simultaneously, the differential microbial biomarkers and protein modules screened in this study lay an important data foundation for developing biomarker panels for dry eye risk prediction.

## Data Availability

The original contributions presented in the study are included in the article/**Supplementary Material**. Further inquiries can be directed to the corresponding author.

## Supplementary material

Supplementary Figure 1 NMDS of microbial beta diversity based on weighted **(a)** and unweighted **(b)** unifrac distances.

Supplementary Figure 2 Relative abundance of major bacterial phyla across the three groups.

Supplementary Figure 3 Random forest classification performance (PG vs. DEG) with varying numbers of microbial features. The line plot shows the model performance, measured by the AUC in both the training set **(a)** and test set **(b)**.

Supplementary Figure 4 Functional enrichment analysis of the top 10 differentially expressed proteins between DEG and PG groups. **(a, b)** Top 10 significantly enriched GO terms for **(a)** upregulated and **(b)** downregulated proteins, categorized into biological process, cellular component, and molecular function. **(c–e)** Top 10 significantly enriched KEGG pathways for **(c)** all differentially expressed proteins, **(d)** upregulated proteins, and **(e)** downregulated proteins. **(f–h)** Top 10 significantly enriched Hallmark gene sets for **(f)** all differentially expressed proteins, **(g)** upregulated proteins, and **(h)** downregulated proteins.

Supplementary Figure 5 Functional enrichment analysis of the top 10 differentially expressed proteins between NDEG and PG groups. **(a, b)** Top 10 significantly enriched GO terms for **(a)** upregulated and **(b)** downregulated proteins, categorized into biological process, cellular component, and molecular function. **(c–e)** Top 10 significantly enriched KEGG pathways for **(c)** all differentially expressed proteins, **(d)** upregulated proteins, and **(e)** downregulated proteins. **(f–h)** Top 10 significantly enriched Hallmark gene sets for **(f)** all differentially expressed proteins, **(g)** upregulated proteins, and **(h)** downregulated proteins.

Supplementary Figure 6 Functional enrichment analysis of the top 10 differentially expressed proteins between DEG and NDEG groups. **(a, b)** Top 10 significantly enriched GO terms for **(a)** upregulated and **(b)** downregulated proteins, categorized into biological process, cellular component, and molecular function. **(c–e)** Top 10 significantly enriched KEGG pathways for **(c)** all differentially expressed proteins, **(d)** upregulated proteins, and **(e)** downregulated proteins. **(f–h)** Top 10 significantly enriched Hallmark gene sets for **(f)** all differentially expressed proteins, **(g)** upregulated proteins, and **(h)** downregulated proteins.

Supplementary Figure 7 GO functional enrichment analysis of the four core protein clusters identified by fuzzy c-means clustering. **(a–d)** display the top 10 significantly enriched Gene Ontology (GO) terms for Cluster 1 through Cluster 4, respectively.

Supplementary Figure 8 Receiver operating characteristic (ROC) curves depicting the classification performance of Random Forest models trained on differentially expressed proteins. **(a)** PG vs NDEG, **(b)** DEG vs PG, **(c)** NDEG vs PG.
