# Supplementary figures and images for: Integrated microbiomic and proteomic profiling reveals distinct ocular surface molecular and microbial landscapes in dry eye after SMILE surgery
Source: Front Cell Infect Microbiol. 2026 Jul 15;16:1858069. doi: 10.3389/fcimb.2026.1858069 (PMC13416856; doi:10.3389/fcimb.2026.1858069)

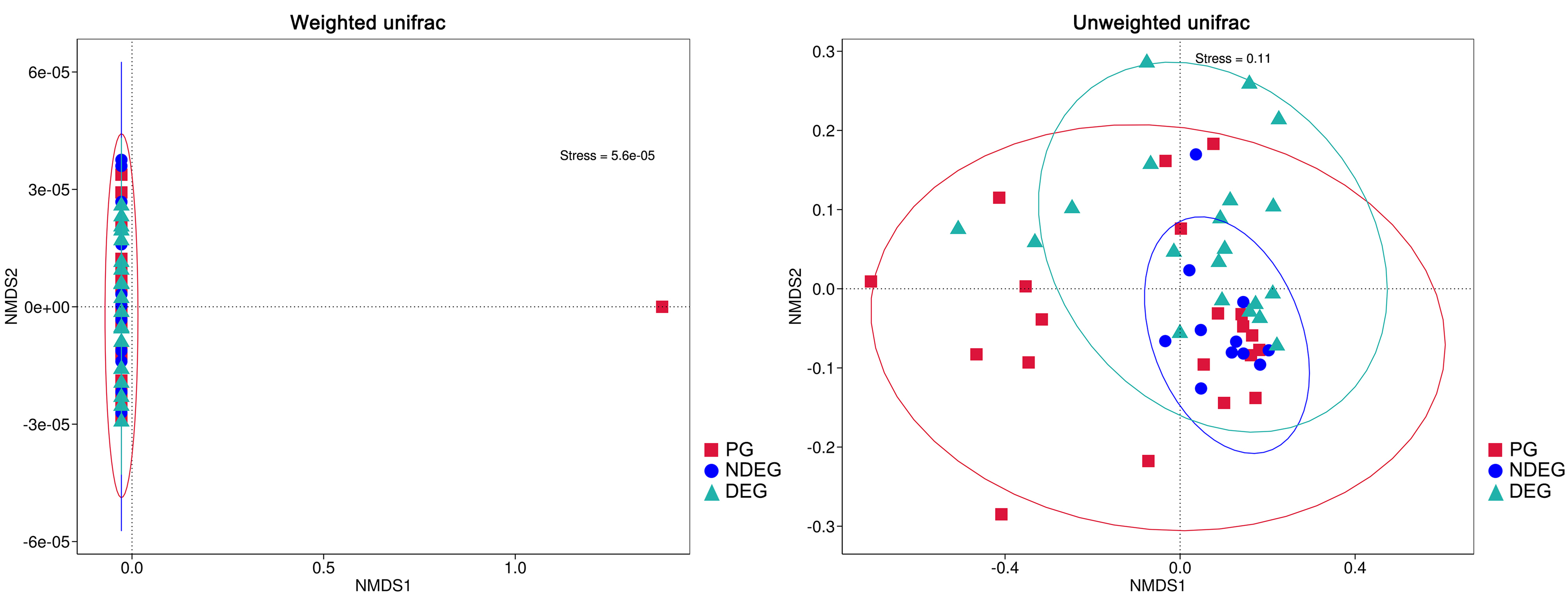

Supplement: Supplementary Figure 1 — NMDS of microbial beta diversity based on weighted (a) and unweighted (b) unifrac distances. [file Image1.jpeg]

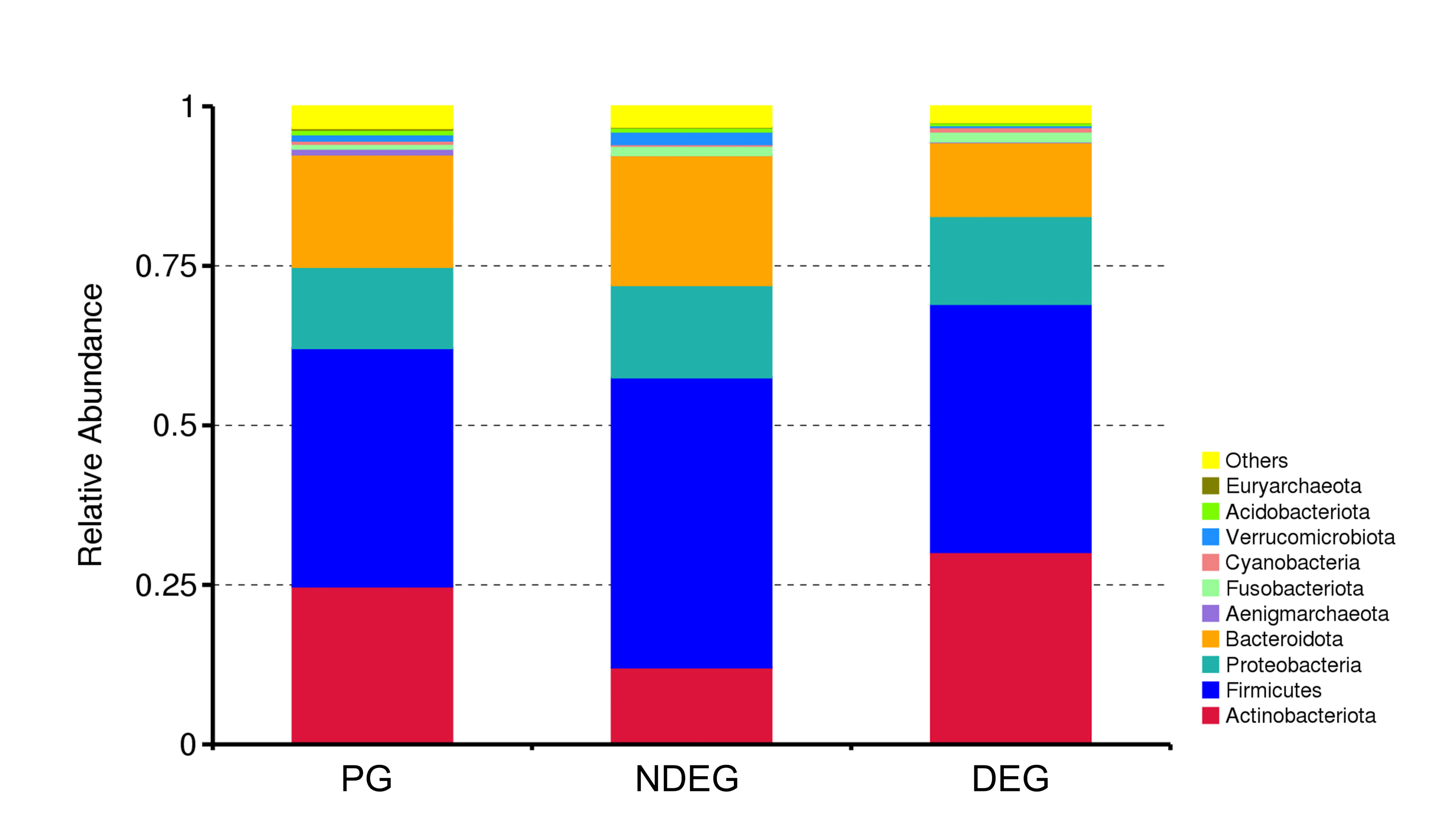

Supplement: Supplementary Figure 2 — Relative abundance of major bacterial phyla across the three groups. [file Image2.jpeg]

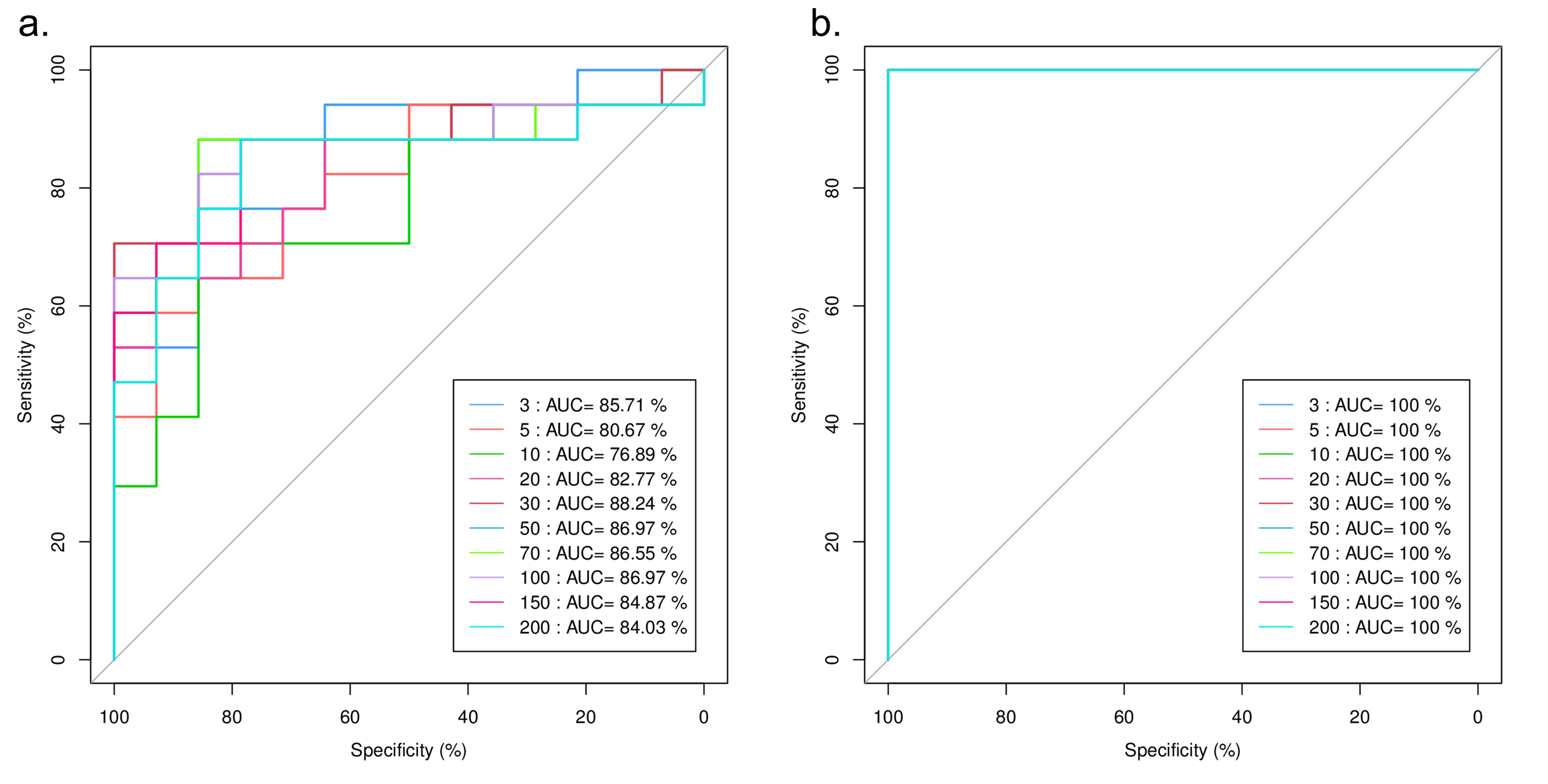

Supplement: Supplementary Figure 3 — Random forest classification performance (PG vs. DEG) with varying numbers of microbial features. The line plot shows the model performance, measured by the AUC in both the training set (a) and test set (b). [file Image3.jpeg]

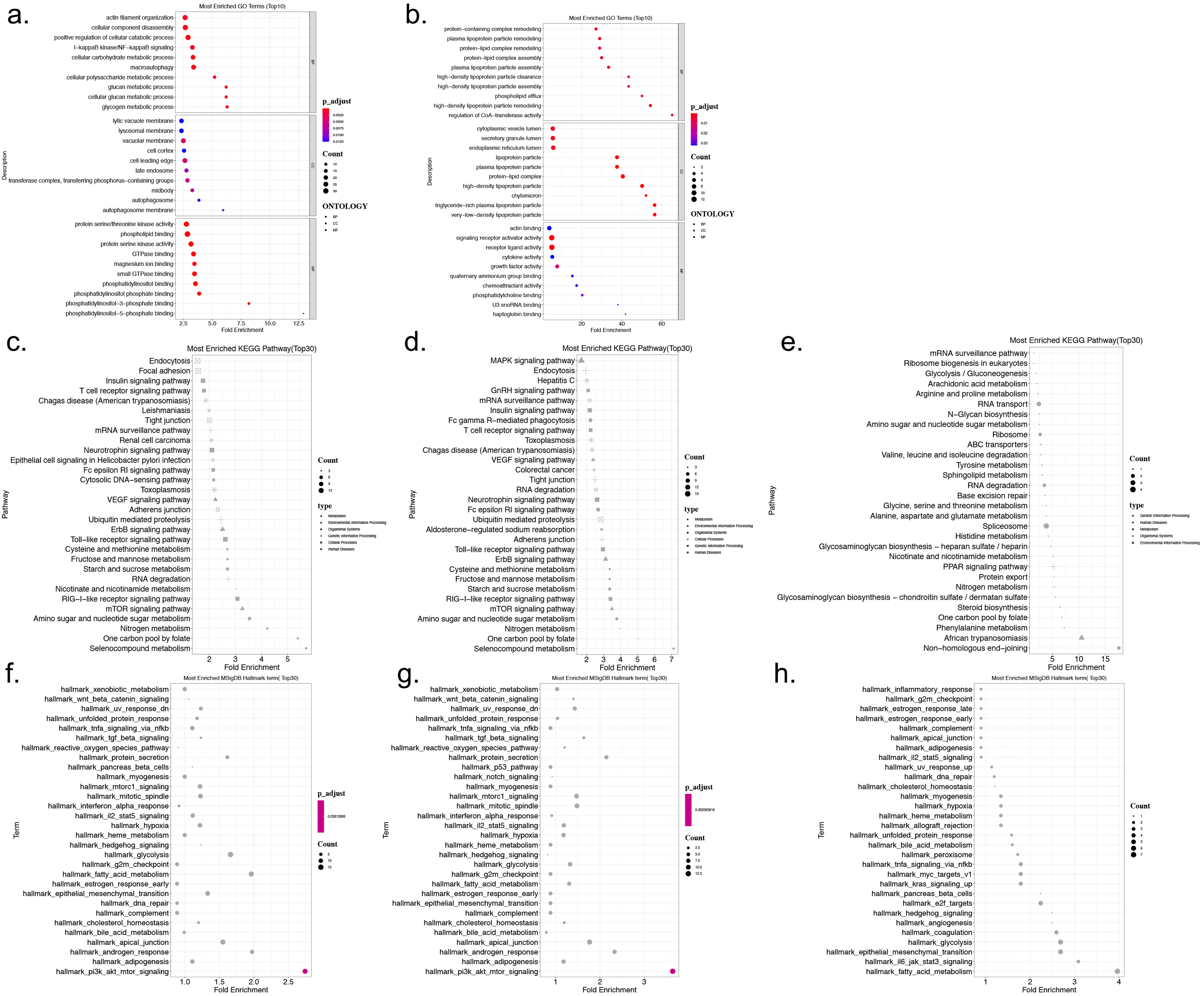

Supplement: Supplementary Figure 4 — Functional enrichment analysis of the top 10 differentially expressed proteins between DEG and PG groups. (a, b) Top 10 significantly enriched GO terms for (a) upregulated and (b) downregulated proteins, categorized into biological process, cellular component, and molecular function. (c–e) Top 10 significantly enriched KEGG pathways for (c) all differentially expressed proteins, (d) upregulated proteins, and (e) downregulated proteins. (f–h) Top 10 significantly enriched Hallmark gene sets for (f) all differentially expressed proteins, (g) upregulated proteins, and (h) downregulated proteins. [file Image4.jpeg]

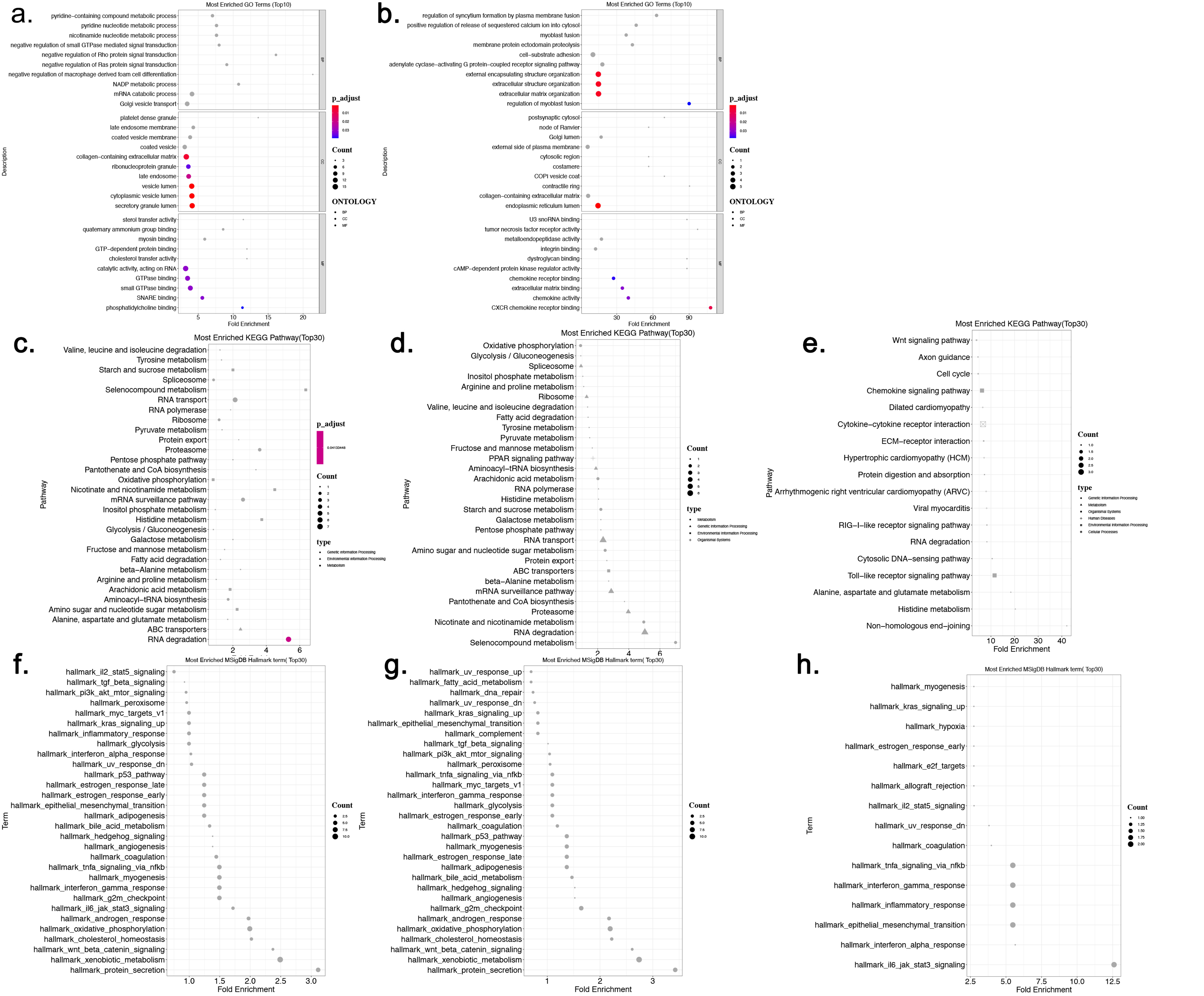

Supplement: Supplementary Figure 5 — Functional enrichment analysis of the top 10 differentially expressed proteins between NDEG and PG groups. (a, b) Top 10 significantly enriched GO terms for (a) upregulated and (b) downregulated proteins, categorized into biological process, cellular component, and molecular function. (c–e) Top 10 significantly enriched KEGG pathways for (c) all differentially expressed proteins, (d) upregulated proteins, and (e) downregulated proteins. (f–h) Top 10 significantly enriched Hallmark gene sets for (f) all differentially expressed proteins, (g) upregulated proteins, and (h) downregulated proteins. [file Image5.jpeg]

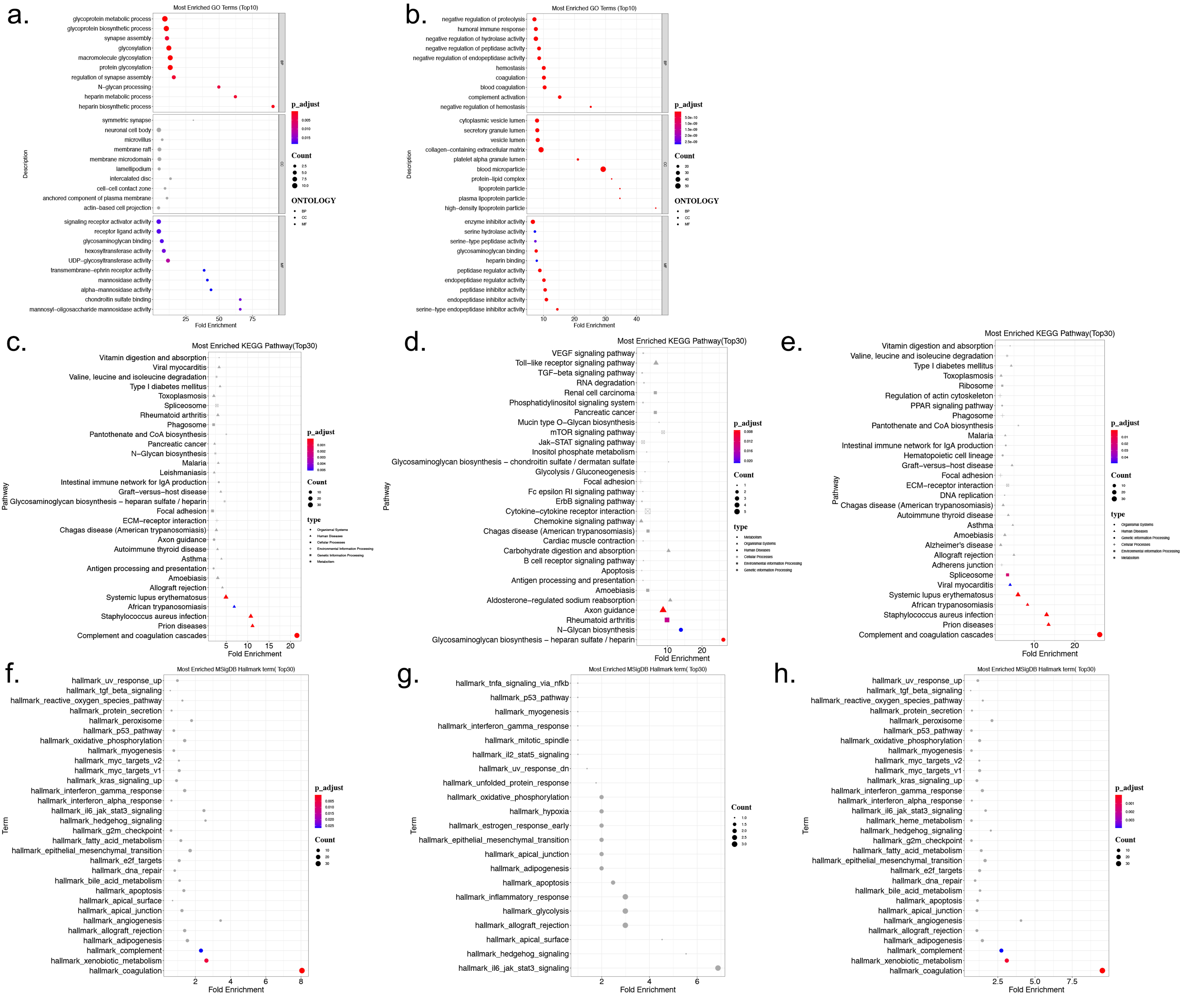

Supplement: Supplementary Figure 6 — Functional enrichment analysis of the top 10 differentially expressed proteins between DEG and NDEG groups. (a, b) Top 10 significantly enriched GO terms for (a) upregulated and (b) downregulated proteins, categorized into biological process, cellular component, and molecular function. (c–e) Top 10 significantly enriched KEGG pathways for (c) all differentially expressed proteins, (d) upregulated proteins, and (e) downregulated proteins. (f–h) Top 10 significantly enriched Hallmark gene sets for (f) all differentially expressed proteins, (g) upregulated proteins, and (h) downregulated proteins. [file Image6.jpeg]

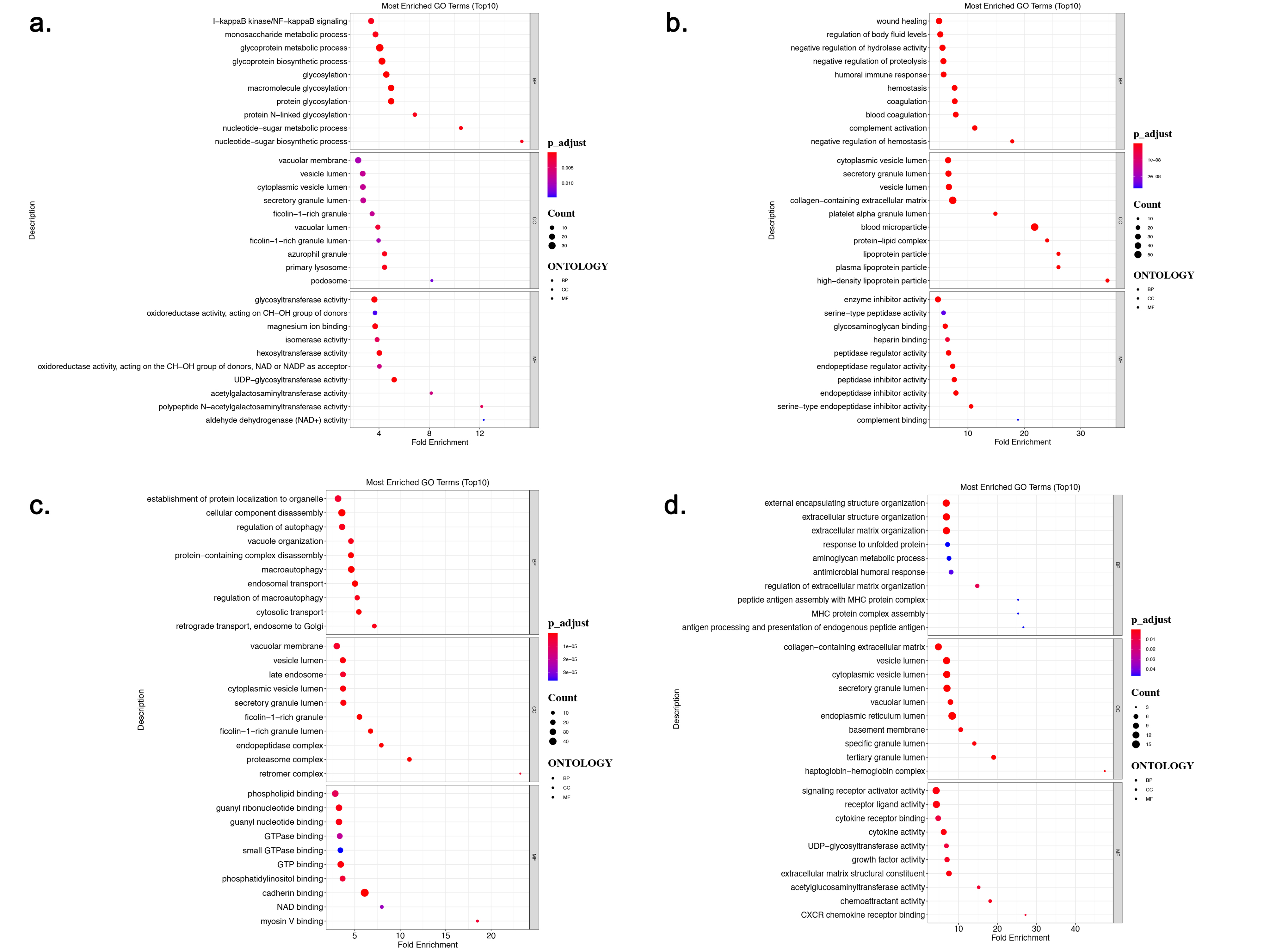

Supplement: Supplementary Figure 7 — GO functional enrichment analysis of the four core protein clusters identified by fuzzy c-means clustering. (a–d) display the top 10 significantly enriched Gene Ontology (GO) terms for Cluster 1 through Cluster 4, respectively. [file Image7.jpeg]

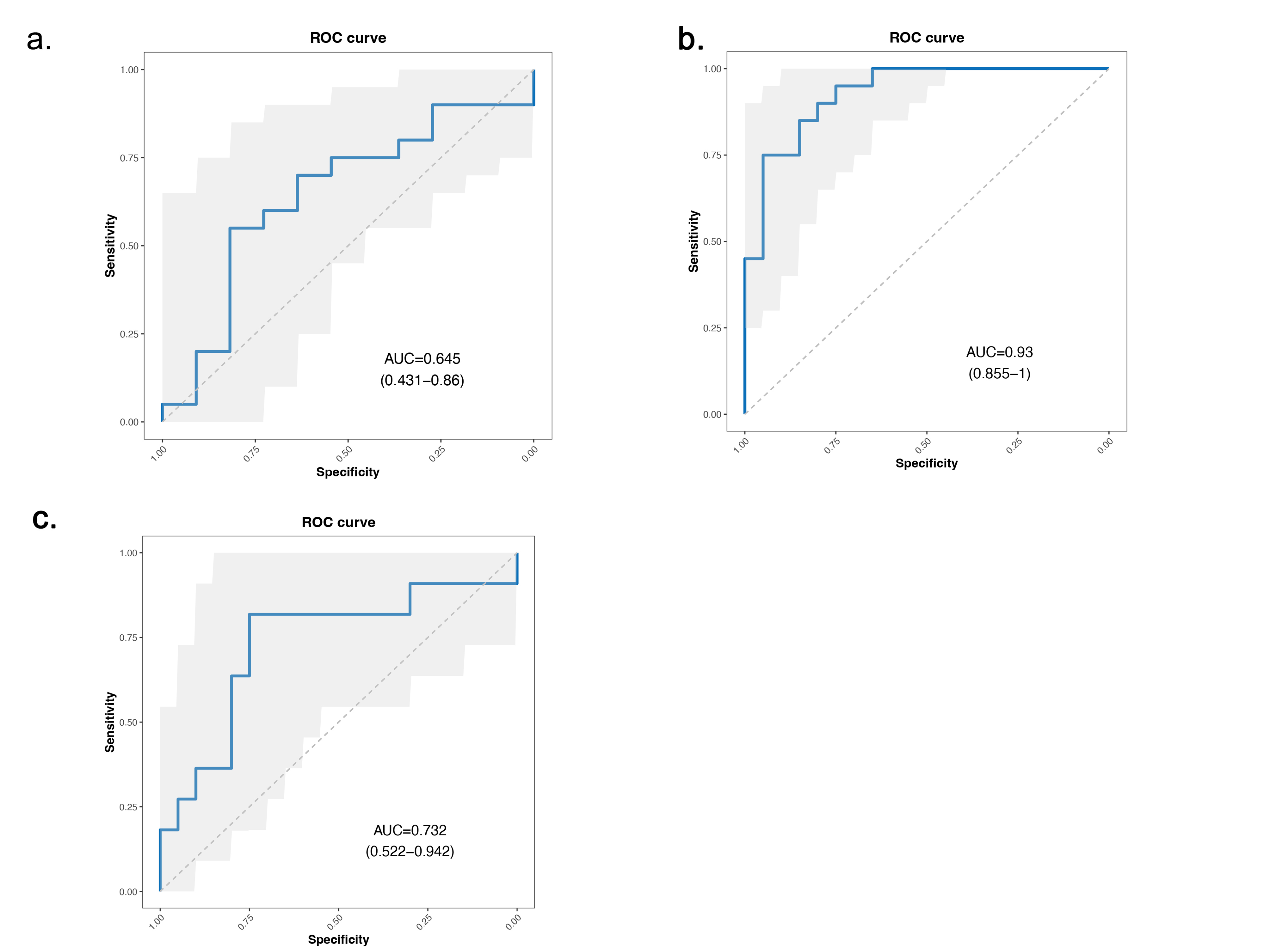

Supplement: Supplementary Figure 8 — Receiver operating characteristic (ROC) curves depicting the classification performance of Random Forest models trained on differentially expressed proteins. (a) PG vs NDEG, (b) DEG vs PG, (c) NDEG vs PG. [file Image8.jpeg]
